# Miscellaneous

**Published:** 1892-06

**Authors:** 


### MISCELLANEOUS.

### COOKING DEPARTMENT AT THE COMING FOOD MANUFACTURERS' EXHIBITION.

An arrangement has been made with Miss Maria Parloa, of Boston, the distinguished lecturer on the art of cooking, whereby she is to take entire charge of that department of the Exposition, to be held at Madison Square Garden, New York, in October next. Miss Parloa will lecture and give practical demonstrations in cooking, every afternoon during the Exposition, in the magnificent Music Hall at the Garden.

Oatmeal as a Drink.—With the season of sunstrokes at hand the following recipe from the pen of the late Dr. Parkes, an eminent English physician, may prove a blessing to workingmen—especially to those who must bear the heat and burden of the day in mill or harvest field. It is said by those who have tried this drink that they could accomplish more work than when using beer, and were physically in better condition.

The proportions are a quarter pound of oatmeal to two or three quarts of water, according to the heat of the day and the work and thirst; it should be well boiled, and then an ounce or one and a half ounces of brown sugar added. If you find it thicker than you like add three quarts of water. Before drinking it shake up the oat meal well through the liquid. In summer drink this cold; in winter hot. You will find it not only quenches thirst but will give you more strength and endurance than any other drink. If you cannot boil it you can take a little oatmeal mixed with cold water and sugar, but this is not so good; always boil it if you can. If at any time you have to make a very long day, as in harvest, and cannot stop for meals, increase the oatmeal to half-pound or even three-quarters pound, and the water to three quarts if you are likely to be very thirsty.

Curious Trees —There are many vegetable wonders in this world of ours. Certain tropical trees furnish clothes as well as food, and the inner bark is smooth and flexible enough to serve as writing paper. The bread tree has a solid fruit, a little larger than a cocoanut which, when cut in slices and cooked, can scarcely be distinguished from excellent bread. The weeping tree of the Canary Islands is wet, even in a drought, constantly distilling water from its leaves, and the wine tree of Mauritius Island furnishes good wine instead of water. A kind of ash in Sicily has a sap which hardens into crude sugar, and is used as such by the natives, without any refining. The product of the wax tree of the Andes resembles bees' wax very closely. Then there is the butter tree of Africa which produces as much as a hundred pounds at once, only to be renewed in a few months. This secretion, when hardened and salted, is difficult to distinguish from fresh, sweet butter. Closely rivalling this is the milk tree of South America, the sap of which resembles rich cow's milk, and is used as such by the natives. China can boast of a soap tree, the seeds of which, when used as soap, produce a strong suds and remove dirt and grease readily. In direct opposition to these useful trees is the man-eating plant of the tropics, which resembles Venus' fly-trap in its nature. It has a short, thick trunk, limed with narrow, flexible barbed spines.

Night Air.—The proofs that night air is not injurious are innumerable. We are told that people who live for the most part of their time in the open air, sleeping under hedges, or under the lee of any kind of shelter, are seldom afflicted with diseased lungs; and the number of soldiers who were cured of incipient consumption and kindred diseases in America, during the war, was legion. They went away as to certain death — not from shells and bullets, but from exposure to "night air." However, they grew hale and hearty, their coughs left them, their chests deepened, their thin cheeks filled out, and those of them who did not fall under the hands of their brethren came home new men. Besides the prejudice against night air, there is another, even more pronounced against draughts. People fear a draught as they fear the plague, and yet no room can ever be purged of foul air without one. Unless there is an ingress and egress of pure air into a room, the atmosphere stagnates like water in a pond. It is not, perhaps, wise for any person who is not used to it to sit directly in the line of a current, although we do not believe there are many colds from draughts ; but all the air of the room will be refreshed in a very few minutes if the draught be reasonably strong.

The Use of Cream.—Very few housekeepers can realize the nutritive value of cream, and understand its superiority to any other solid fats, in permitting the gastric juice to mix with it in the most perfect manner, and in this way aiding and hastening digestion. It is invaluable in the case of invalids, for it serves as nutriment in a very available form. It is superior to butter, because it contains more volatile oil than butter made from it. It is frequently ordered by physicians for persons consumptively inclined, for those with feeble digestions, for aged persons, and for those who suffer from impaired circulation, cold feet, and who feel chilly from want of nutriment. No other article of food gives such satisfactory results. It is, however, expensive in large cities, and difficult to get fresh and sweet. On a farm, however, it can be had in its sweetness, and it can be freely used. Whipped, it can be served in dozens of ways, with fresh or stewed fruits, as an accompaniment to cake, puddings, and the like, while cream can be drunk nearly as freely as milk. For use in whipping, it should be thick and sweet, while for drinking it can be used after the milk has stood, at the most, but a few hours over night.

Insomnia and Its Cure.—A correspondent, who asks for a cure for insomnia, is advised to drop all anodynes and opiates. Subjective mental exercise of the right kind, is far more efficacious than any drug, or even hot milk. Upon retiring, resolutely bar out of mind the material environment and rivet the thought tenaciously to some *one* lofty spiritual conception. Persist in this, even if at first the exercise seems purely mechanical. The power of mental concentration will grow. It is a fact that this course will often cure chronic and severe insomnia in a few weeks. There is nothing strange or miraculous about it, but when understood it is seen to be natural, reasonable and scientific, Those who have not looked into this subject will be surprised at the rapidly developing power of mind in this direction when faithfully tried. It amounts to a self-mental treatment, and the nerves will respond in a reasonable time. The cure will be permanent, and the self-control which is gained will be found very valuable.

Ear-rings.—If you and your sister are bent on piercing your ears, you had far better go to a good jeweller's and have the operation performed properly, as although I have heard of persons doing it for each other with a very coarse needle or fine stiletto and a cork, still it is most unwise. But I should strongly advise you to forego your intention. The custom has nothing to recommend it, and now has not even the merit of being fashionable, for out of twenty really well dressed and well known women nineteen have no earrings, and the twentieth probably only wears them because she possesses valuable diamonds, which she is anxious to exhibit. It is asserted, but not on particularly good grounds, that in certain cases of weak eyes the counter-irritation set up by the slight wounds in the ears does some good for a time; but it is most assuredly of not the least permanent benefit. If, however, you are determined to wear these ornaments, choose the smallest possible; tiny round knobs of gold or silver are best; long earrings are considered exceedingly vulgar.

THE HERB OF PROPHECY.—M. Carrera, deputy of Oaxaca, has taken to the city of Mexico a plant which is known to grow only in Mixteca, called the "herb of prophesy" by the natives. Devotees of this weed take it much in the same manner that cocoa leaves are taken by those addicted to the habit. In a few moments after a dose of it has been taken a sleep is produced similar in all respects to, and, it might be said, identical with, the hypnotic state. When under its influence the sleeper is completely insensible, but will answer with closed eyes all questions put to him.

It is further said of this wonderful plant that the pathologic state induced on whomsoever partakes of the herb brings with it a kind of prophetic gift and second sight. One who has taken this herb loses his will even more completely than does the person who is in the hypnotic state, and is so thoroughly under the control of any voice that he would shoot or stab himself at any moment if commanded to do so. When one regains his senses after being under the influence of the "prophetic herb" he remembers nothing of what he has done when in the trance.

A GIANT APPLE TREE.—The largest apple tree in New England, and probably in the world, is in the northwestern part of Cheshire, Conn. Its age can be traced by a family tradition to 140 years, at least, and it may be twenty or twenty-five years older. It is, at the presenttime, of symmetrical shape; the trunk is nearly round, without a scar or blemish on it; there are eight large branches; five of them have been in the habit of bearing one year, and the remaining three the next. From the five branches 110 bushels of apples have been gathered. The tree measures 13 feet 8 inches around, is 60 feet high, aud the branches spread 100 feet.

WASHING CHILDREN'S FACES.—Care should always be observed in washing children's faces not to let the soap get in the eyes. A physician writes : I think it cruel to allow the face and eyes to be washed over with soap in the coarse and rough way in which I have often seen it done. Some nurses appear to take a sort of morbid delight in its employment in this way. Even to an adult, soap in the eyes is a very painful ordeal to go through ; in the end it inevitably produces chronic, sometimes acute ophthalmia. Children should be spared this barbarity. In washing children's faces with soap a fine flannel, a sponge, or the corner of a towel should be used.

DIVORCE.—There are more divorces granted in the United States than in all the rest of the Christian world put together. Americans are very discriminative —after marriage.

